# Supplementary material for: Nicotinamide Riboside Augments Human Macrophage Migration via SIRT3-Mediated Prostaglandin E2 Signaling
Source: Cells. 2024 Mar 5;13(5):455. doi: 10.3390/cells13050455 (PMC10931126; doi:10.3390/cells13050455)
Supplement: Supplementary file 1 [file cells-13-00455-s001.zip › cells-2850095-supplementary.pdf]

Supplementary Figures:

Nicotinamide Riboside Augments Human Macrophage Migration via SIRT3-Mediated Prostaglandin E2 Signaling

Jing Wu [1], Maximilian Bley [1], Russell S. Steans [1], Allison M. Meadows [1,2], Rebecca D. Huffstutler [3], Rong Tian [4], Julian L. Griffin [2,5] and Michael N. Sack [1,3,*]

[1] Laboratory of Mitochondrial Biology and Metabolism, National Heart, Lung, and Blood Institute, National Institutes of Health, Bldg. 10-CRC, Room 5-3342, 10 Center Drive, Bethesda, MD 20892, USA; jing.wu@nih.gov (J.W.); maximilian.bley@med.uni-heidelberg.de (M.B.); russell.steans@nih.gov (R.S.S.); allisonm.meadows@gmail.com (A.M.M.)

[2] Department of Biochemistry, Cambridge University, Cambridge CB2 1QW, UK; jules.griffin@abdn.ac.uk

[3] Cardiovascular Branch, National Heart, Lung, and Blood Institute, National Institutes of Health, Bethesda, MD 20892, USA; rebecca.huffstutler@nih.gov

[4] Mitochondria and Metabolism Center, Department of Anesthesiology and Pain Medicine, University of Washington School of Medicine, Seattle, WA 98195, USA; rongtian@uw.edu

[5] The Rowett Institute, School of Medicine, Medical Sciences and Nutrition, Foresterhill Campus, Aberdeen AB25 2ZD, UK

* Correspondence: sackm@nih.gov

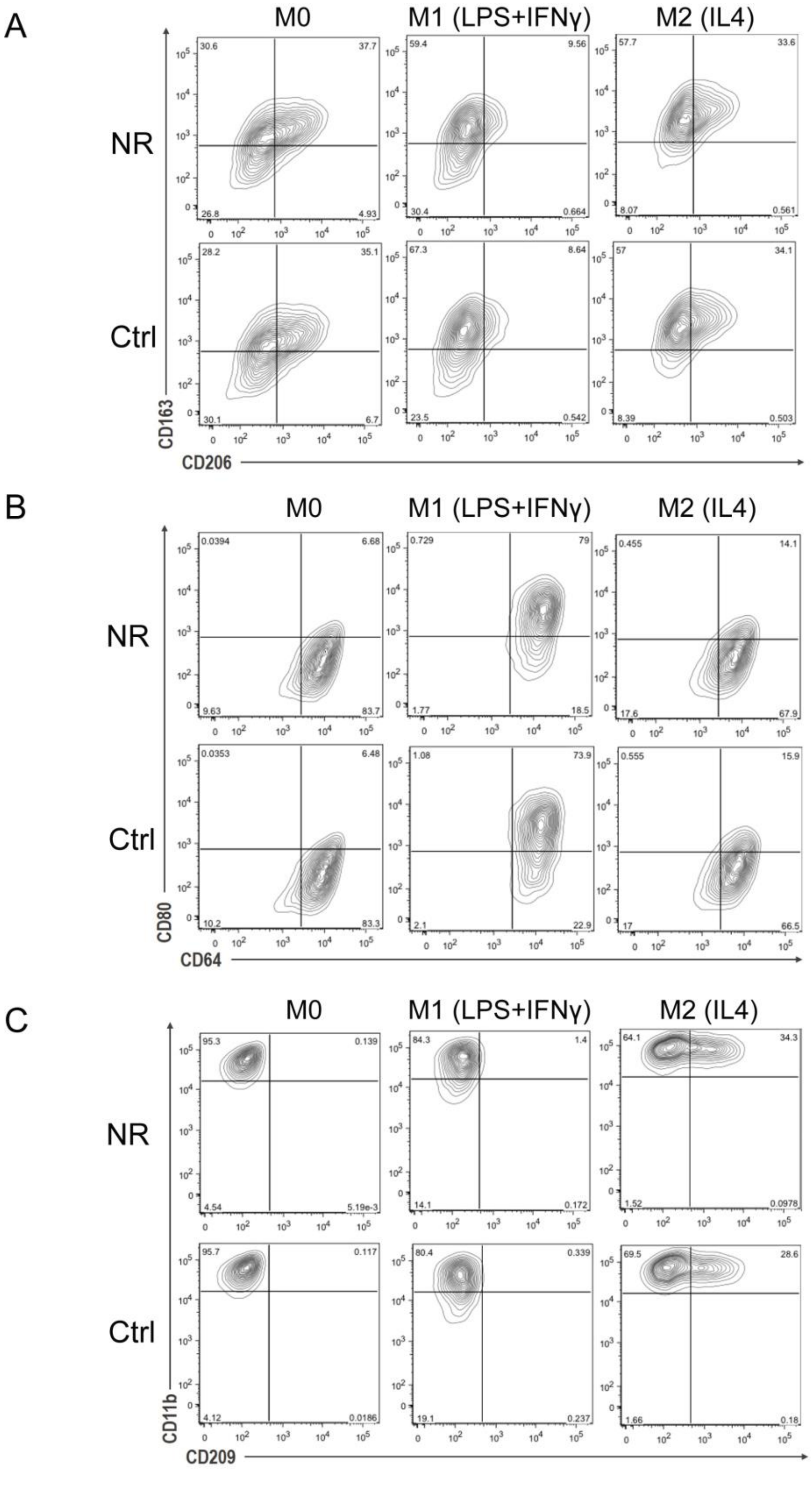
A
M0
M1 (LPS+IFNγ)
M2 (IL4)
NR
Ctrl
CD163
CD206
30.6
37.7
26.8
4.93
59.4
9.56
30.4
0.664
57.7
33.6
8.07
0.561
28.2
35.1
30.1
6.7
67.3
8.64
23.5
0.542
57
34.1
8.39
0.503
B
M0
M1 (LPS+IFNγ)
M2 (IL4)
NR
Ctrl
CD80
CD64
0.0394
6.68
9.63
83.7
0.729
79
1.77
18.5
0.455
14.1
17.6
67.9
0.0353
6.48
10.2
83.3
1.08
73.9
2.1
22.9
0.555
15.9
17
66.5
C
M0
M1 (LPS+IFNγ)
M2 (IL4)
NR
Ctrl
CD11b
CD209
95.3
0.139
4.54
5.19e-3
84.3
1.4
14.1
0.172
64.1
34.3
1.52
0.0978
95.7
0.117
4.12
0.0186
80.4
0.339
19.1
0.237
69.5
28.6
1.66
0.18

Figure S1. Flow cytometric analysis of polarization with NR supplementation. Representative contour plots showing CD163 and CD206 expression **(A)**, CD64 and CD80 expression **(B)**, CD11b and CD209 expression **(C)** in 3 different types of human MDMs: M0, M1(LPS+IFNγ), and M2 (IL4) treated with either vehicle (Ctrl, bottom panels) or NR (top panels). The gates represent surface marker expression as a percentage to living cells. These plots confirmed the polarization and there was no visible shift of different populations with or without NR exposure.

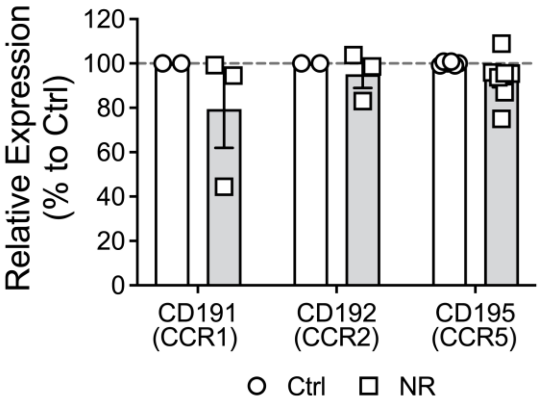

Figure S2. Flow cytometric analysis of canonical chemokine receptors with NR supplementation.

Quantification of relative surface expression of CCR1 (CD191), CCR2 (CD192) and CCR5 (CD195) in human M1 MDMs treated with vehicle (Ctrl) or NR for 48hr. Data were analyzed using unpaired two-tailed Student's *t*-test, and represented as mean ± SEM.

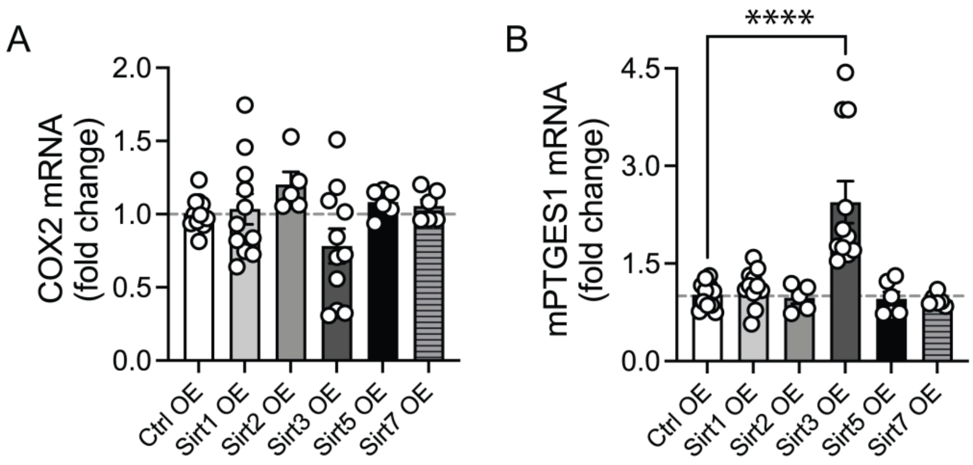

Figure S3. Overexpression of Sirtuin family members on transcript levels of PGE2 synthesis genes.

Quantitative RT-PCR analysis of COX-2 **(A)** and mPTGES-1 **(B)** transcripts in activated human monocytes (20ng/mL LPS for 8hr) transfected with either empty vector (Ctrl OE) or different Sirtuin-expression plasmids (n=5-11 replicates from 2-4 independent experiments). Data normalized to 18S rRNA and represented as mean ± SEM. One-way analysis of variance (ANOVA) with Dunnett's multiple comparisons test. ****p<0.0001.

Table S1: Human Macrophage Surface Markers and Flow Cytometry Antibodies used in this study.

Cluster of Differentiation	Catalog No.	Company	Fluorophore	Clone	Highest Expression in
CD64	555527	BD Bio	FITC	10.1	M1
CD80	561135	BD Bio	PECy7	L307.4	
CD197	353204	Biolegend	PE	G043H7	
CD11b	555388	BD Bio	PE	Mac-1	M2
CD209	330108	BioLegend	APC	9E9AS	
CD163	333616	BioLegend	BV605	GH1/61	M0/M2
CD206	564062	BD Bio	BV421	19.2	
CD14	561383	BD Bio	APC	M5E2	
CD191	362907	BioLegend	APC	5F10B29	
CD192	357208	BioLegend	APC	K036C2	
CD192	357213	BioLegend	BV605	K036C2	
CD195	565224	BD Bio	BUV395	2D7/CCR5	
Aqua	L34966	Invitrogen			
DAPI	564907	BD Bio			
FcγR	564220	BD Bio			
